# Supplementary figures and images for: Evidence of long-term allocentric spatial memory in the Terrestrial Hermit Crab Coenobita compressus
Source: PLoS One. 2023 Oct 26;18(10):e0293358. doi: 10.1371/journal.pone.0293358 (PMC10602228; doi:10.1371/journal.pone.0293358)

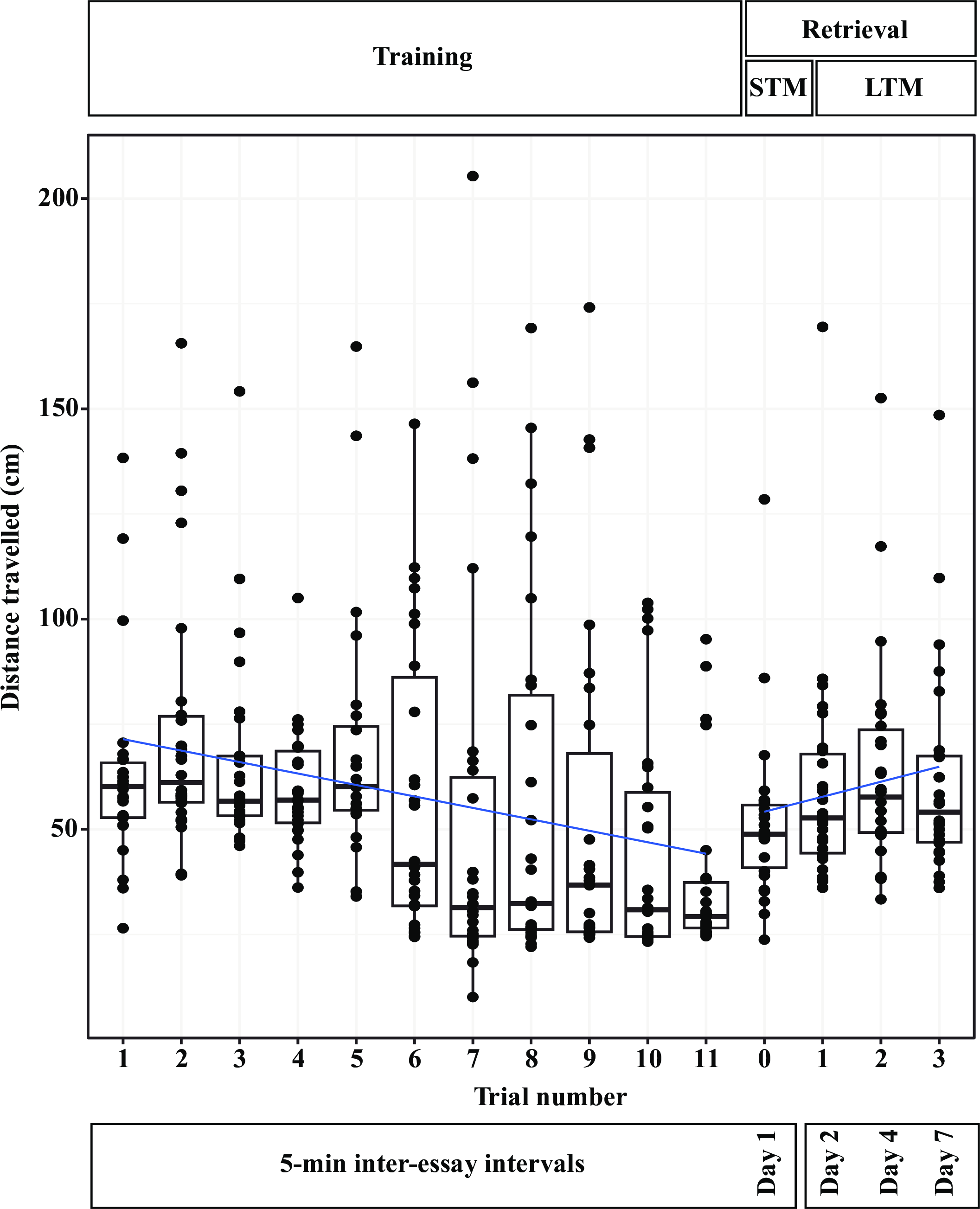

Supplement: S1 Fig — Solid blue lines represent a function describing the training and retrieval phases of n = 26 hermit crabs as modeled by a generalized linear mixed-effect model (GLMM). Points (closed circles) represent distance for each crab. Median and interquartile ranges distance in cm are presented. Decrease in distance to reach the target during training and increase during short- and long-term memory retrieval followed the expected trend but was non-significant. (TIF) [file pone.0293358.s001.tif]
